# Effect of capsular tension ring implantation on capsular stability after phacoemulsification in patients with weak zonules: a randomized controlled trial. CTR implantation in cataract patients with weak zonules

**DOI:** 10.1186/s12886-020-01772-8

**Published:** 2021-01-07

**Authors:** Shangfei Yang, Hui Jiang, Kailai Nie, Liwen Feng, Wei Fan

**Affiliations:** Department of Ophthalmology, West China Hospital of Sichuan University, Chengdu, 610041 Sichuan Province China

**Keywords:** Capsular tension ring, Weak zonules, Pars plana vitrectomy, Severe myopia, Phacoemulsification, Cataract

## Abstract

**Background:**

The use of capsular tension ring (CTR) implantation to treat cataract patients with weak zonules is still controversial. The aim of this study was to examine the effects of CTR implantation on capsular stability after phacoemulsification in patients with weak zonules, especially patients who have undergone pars plana vitrectomy (PPV) or those who suffer from severe myopia.

**Methods:**

A total of 42 patients who underwent phacoemulsification and received an intraocular lens (IOL) were randomized to undergo CTR implantation or not. The control and CTR groups were compared in terms of uncorrected distant visual acuity (UDVA), best corrected distant visual acuity (BCDVA), refractive prediction error, the area of anterior capsulorhexis, and IOL inclination angle. Follow-up visits were conducted postoperatively at 1 day, 1 week, 1 month and 3 months. Subgroup analyses were performed based on PPV and severe myopia.

**Results:**

Surgery significantly improved UDVA and BCDVA to similar extents in CTR and control patients, and refraction prediction error was similar between the two groups at all follow-up times. At 3 months after surgery, the area of anterior capsulorhexis was significantly larger in CTR patients than in controls (*p* = 0.0199). These differences were also significant between the subgroups of patients with severe myopia. Vertical IOL inclination was less within CTR groups at 3 months after surgery, especially in patients with severe myopia (*p* = 0.0286). At 1 week postoperatively, the proportion of individuals whose posterior lens capsule that had completely adhered to the posterior IOL surface was significantly higher among CTR patients (*p* = 0.023). No serious surgical complications were observed.

**Conclusion:**

CTR implantation can benefit cataract patients with weak zonules by maintaining the shape of the capsular bag, reducing capsule shrinkage and stabilizing IOL inclination.

**Trial registration:**

Chinese Clinical Trial Registry ChiCTR-INR-17011217, date of registration April 22, 2017, prospectively registered.

## Background

Zonular fibers connect the ciliary body to the equator of the lens and maintain the position of the lens as well as adjust its curvature. Zonules are considered weak if zonular fibers are looser than normal, making them more susceptible to damage or rupture, or if they have already been ruptured. Zonular weakness makes the lens unstable, which can complicate intraocular procedures such as cataract surgery and increase risk of intraocular lens (IOL) dislocation [1]. IOL dislocation is a severe complication after cataract surgery, and it necessitates additional surgery to replace the IOL using different techniques, which can be very invasive for the eye.

A number of factors can predispose individuals to weak zonules, such as vitrectomy [2–6], high myopia [4, 7–9], ageing [10], pseudoexfoliation syndrome [5, 11, 12], retinal pigment degeneration [13], Marfan syndrome [14], and eye injury [4, 5]. In Chinese patients, vitrectomy and strong myopia are the frequent causes of a weak zonule, so the present study focused on patients who had undergone pars plana vitrectomy (PPV) or who had severe myopia. PPV necessarily damages and weaken zonular fibers near the pars plana [8, 9, 15, 16]. People with strong myopia also have longer zonular fibers because the axial length is longer, and the wall of the eyeball is thinner. Therefore, these patients are at increased risk of a loosened capsular bag, unstable anterior chamber, and lens dislocation during cataract surgery. The chance of capsular shrinkage is also higher after surgery [6, 17, 18]. Capsular shrinkage increases tension on zonular fibers, further lengthening and weakening them [1, 4, 6].

Patients with severe zonular fiber rupture typically undergo extracapsular cataract extraction, intracapsular cataract extraction, or pars plana lensectomy, and the IOL can be implanted in the anterior chamber or sulcus posterior chamber, or it can be sutured to the iris or sclera [19]. However, these surgical procedures are more difficult and time-consuming, and carry high risk of post-operative complications [20–22].

There is no effective treatment for zonular fiber relaxation or mild rupture. The most promising approach is the use of a capsular tension ring (CTR) together with small-incision phacoemulsification and in-the-bag posterior chamber IOL implantation. The CTR maintains the shape of the capsular bag, balances the tension in zonular fibers, and decreases the risk of capsular shrinkage and IOL decentration or dislocation [23–30]. While CTR has been used to treat focal zonular rupture or diffuse zonulopathy by stretching out the capsular bag or facilitating scleral suture fixation [7, 23–35], it is unclear whether the capsular bag and IOL can remain stable after CTR implantation during cataract surgery in patients with strong myopia [7, 23, 35]. In addition to these uncertainties, the effects of CTR on cataract surgery in patients with an abnormal zonule after PPV are unclear. Therefore, the current study analyzed the effects of CTR on capsular bag changes and complications after cataract surgery, in patients with weak zonules, particularly patients who have undergone PPV or who have strong myopia.

## Methods

### Patient enrollment

Consecutive cataract patients who were referred to the Department of Ophthalmology at West China Hospital of Sichuan University between December 2017 and December 2018 were eligible for enrollment if (1) phacoemulsification was proposed as a treatment; (2) patients had an axial length > 28 mm, or more than 3 months had passed since PPV; (3) patients had weak zonules based on an average zonular length > 0.7 mm, as measured by ultrasound biomicroscopy; and (4) patients were eligible for phacoemulsification alone or with CTR implants. Exclusion criteria included: (1) zonular issues caused by pseudoexfoliation syndrome, uveitis, retinitis pigmentosa, trauma, other intraocular surgeries than PPV, or connective tissue disorders; (2) zonular rupture > 90°; and (3) unsuitability for CTR implants. Only data on the first operated eye were included in this study, regardless of whether patients underwent operations in both eyes. Patients were randomly assigned to phacoemulsification alone or with CTR implants based on permuted-block randomization by an optometrist who was not involved in patient selection or surgery. A computer program for randomization that provided random permuted blocks was used by the optometrist. Using a two-sided α = 0.05, β = 0.2, δ (expected difference) = 0.17, and σ (standard deviation) = 0.2, we calculated a minimum sample of 21 patients per group. Assuming an expected dropout rate of 10%, 23 patients in each group were needed.

All patients were given thorough information about the surgery and possible complications, and they were not blinded to treatments. Written informed consent was obtained from all patients for their anonymized clinical data to be analyzed and published for research purposes. This study was approved by the Ethics Committee of the West China Hospital of Sichuan University (Chengdu, China) and registered in the Chinese Clinical Trial Registry (ChiCTR-INR-17011217).

### Preoperative eye examination

**Fig. 1:**
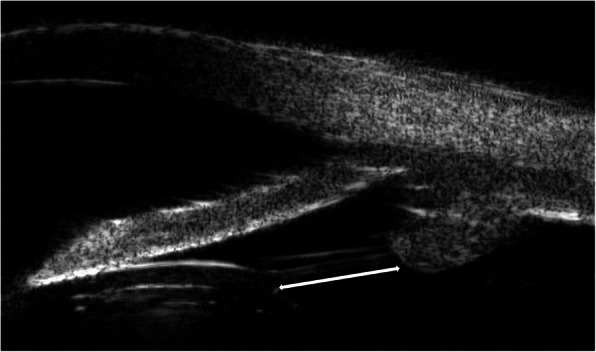
Micrographs showing zonular length by ultrasound biomicroscopy. Arrows indicate zonule

Uncorrected distant visual acuity (UDVA) and best corrected distant visual acuity (BCDVA) of all patients were recorded using a Snellen chart. Visual acuity values were converted to logarithms of the minimum angle of resolution (logMAR) for further analysis. Refractive prediction error was calculated by measuring the difference between the preoperative refractive target and actual postoperative refraction in diopters (D) after surgery. Intraocular pressure was estimated using non-contact tonometry before pupillary dilation, while axial length of the eye was measured using optical biometry (IOL Master 500, Zeiss, Oberkochen, Germany). Zonular weakness was considered if there was an abnormal zonular length, as measured using an ultrasound biomicroscope. Zonular length was reported as an average of four measurements made at 3, 6, 9 and 12 o’clock. These measurements were made between the mid-point of the ciliary process and the end of zonular fibers at the lens equator (Fig. 1). Preoperative eye examinations for all patients were conducted by the same investigator.

### Surgical procedures and IOL implantation

All enrolled patients received phacoemulsification and IOL implantation (Akreos MI60, Bausch & Lomb, Rochester, NY, USA). A 2.0-mm corneal incision was made, a 5.0–5.5 mm continuous curvilinear capsulorhexis (CCC) was performed and the IOL was implanted using a Stellaris System (Bausch & Lomb, Rochester, NY, USA). Patients in the intervention group underwent CTR implantation (ACPi-11, Bausch & Lomb, Rochester, NY, USA) before IOL implantation. All surgical procedures were performed by an experienced surgeon (Wei Fan).

The IOL used in this study was an Akreos MI60 IOL, composed of hydrophilic acrylic with 26% water content, with a total length of 10.5–11 mm and an optic diameter 5.6–6.2 mm, depending on the dioptric power. According to the manufacturer, this IOL has a neutral aspheric optic designed to aid image transmission, even during decentration or tilting. Additionally, this IOL has four haptics designed to resist vitreous pressure and provide anteroposterior stability, thus preventing pseudoaccommodation. The thin haptics provide four zones for capsule sealing around the optic, promoting early and stable centration. The progressive resistance of the haptics is designed to prevent capsular bag contraction and optic displacement. The 10° haptic angle pushes the IOL optics backward. This angle and the 360° square-edged design help prevent PCO [36].

### Postoperative eye examination

Postoperative eye examinations of all patients were performed by the same ophthalmologist and optometrist at 1 day, 1 week, 1 month and 3 months after the surgery. Examination parameters were the same as in the preoperative eye examination. Postoperative complications, if any, were also recorded. Micrographs of anterior continuous curvilinear capsulorhexis and IOL optics were taken using a slit-lamp camera, and areas of anterior capsulorhexis were analyzed by Image J (National Institutes of Health, Bethesda, MD, USA). The position of the IOL and the attachment ratio between the posterior lens capsule and posterior surface of the IOL were evaluated using anterior segment optical coherence tomography (Carl Zeiss Meditec, Jena, Germany). IOL inclination angle, defined as the angle between the posterior surface of the iris and the anterior surface of the IOL, was measured both vertically and horizontally [36]. The angle was measured from micrographs using Adobe PDF Editor (San Jose, CA, USA).

### Statistical analysis

All data were analyzed using SAS 9.4 (IBM, Armonk, NY, USA). Continuous data were presented as mean ± standard deviation and categorical data as number (percentage). For continuous data involving repeated measurements, inter- and intra-group differences were assessed for significance using repeated measurement variance analysis. For categorical data, inter-group differences were assessed using the chi-squared test. All statistical tests were two-sided, and *p* < 0.05 indicated a significant difference.

## Results

### Patient characteristics

A total of 42 patients (21 men) were enrolled in the study and randomized into a CTR group [T (total)-CTR, *n* = 22, 12 men] and control group (T-CON, *n* = 20, 9 men). Subgroup analyses within these two groups were performed based on whether patients suffered from zonular problems because of PPV (P-CTR vs. P-CON) or myopia (M-CTR vs. M-CON). Patients showed the following primary vitreo-retinal diseases: retinal detachment, vitreous hemorrhage, macular pucker, and macular hole. Average length of zonules was (1.08 ± 0.28) mm in the T-CTR group and (1.03 ± 0.19) mm in the T-CON group, and the two values were not statistically different (Table 1).

**Table 1:** Baseline characteristics of patients

Group	n	Sex		Age (yr)	AL (mm)	Length of zonules (mm)
		Male	Female			
T-CTR	22	12 (54.5%)	10 (45.5%)	59.35 ± 12.15	28.32 ± 3.65	1.08 ± 0.28
T-CON	20	9 (45.0%)	11 (55.0%)	61.35 ± 9.35	27.91 ± 3.11	1.03 ± 0.19
* t*				−0.526	0.457	0.628
* p*		0.379		0.605	0.653	0.534
P-CTR	11	6 (54.5%)	5 (45.5%)	59.36 ± 10.99	25.36 ± 1.93	1.01 ± 0.21
P-CON	11	4 (36.4%)	7 (63.4%)	59.36 ± 10.51	26.05 ± 2.67	1.00 ± 0.21
* t*				0.000	−0.694	0.152
* p*		0.335		1.000	0.503	0.881
M-CTR	11	6 (54.5%)	5 (45.5%)	60.27 ± 11.16	30.76 ± 2.36	1.13 ± 0.34
M-CON	9	5 (55.6%)	4 (44.4%)	61.77 ± 9.57	30.19 ± 1.86	1.06 ± 0.18
* t*				−0.916	0.560	0.413
* p*		0.658		0.387	0.591	0.684

*AL* axial length, *T* Total group, *P* post-PPV, *M* myopia, *CTR* capsular tension ring group, *CON* control group

### Vision acuity

**Fig. 2:**
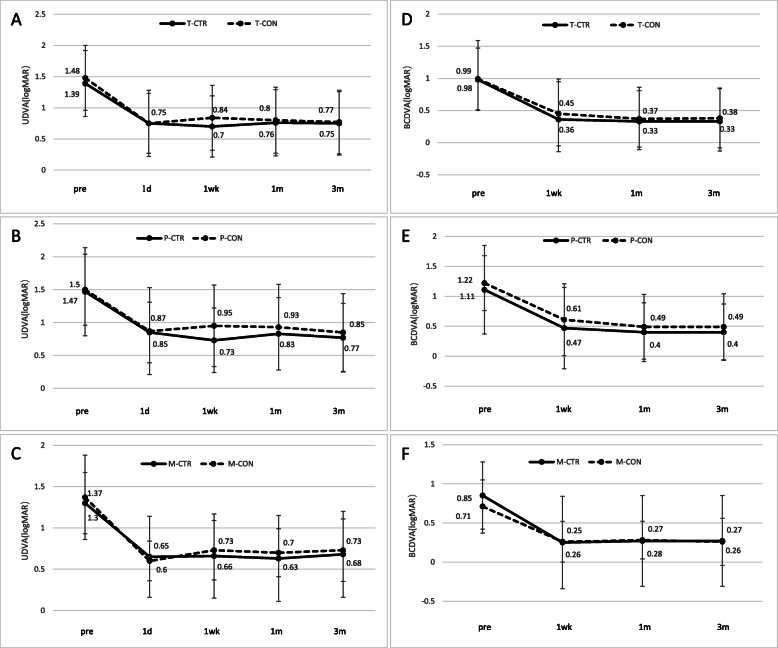
Visual acuity comparison at baseline, 1 week, 1 month, and 3 months after surgery. Comparison of uncorrected distant visual acuity between (**a**) all CTR and control patients, (**b**) the subgroups of CTR and control patients with PPV, and (**c**) the subgroups of CTR and control patients with myopia. Comparison of best corrected distant visual acuity between (**d**) all CTR and control patients, (**e**) the subgroups of CTR and control patients with PPV, and (**f**) the subgroups of CTR and control patients with myopia. T, Total group; P, post-PPV; M, myopia; CTR, capsular tension ring group; CON, control group

UDVA did not differ significantly between CTR and control patients overall (Fig. 2a) or in the subgroups with PPV (Fig. 2b) or severe myopia (Fig. 2c) at any of the time points examined. Similar results were obtained for BCDVA (Fig. 2d-f). In both CTR and control patients, UDVA and BCDVA were significantly better after the operation than before.

### Refractive prediction error (RPE)

**Fig. 3:**
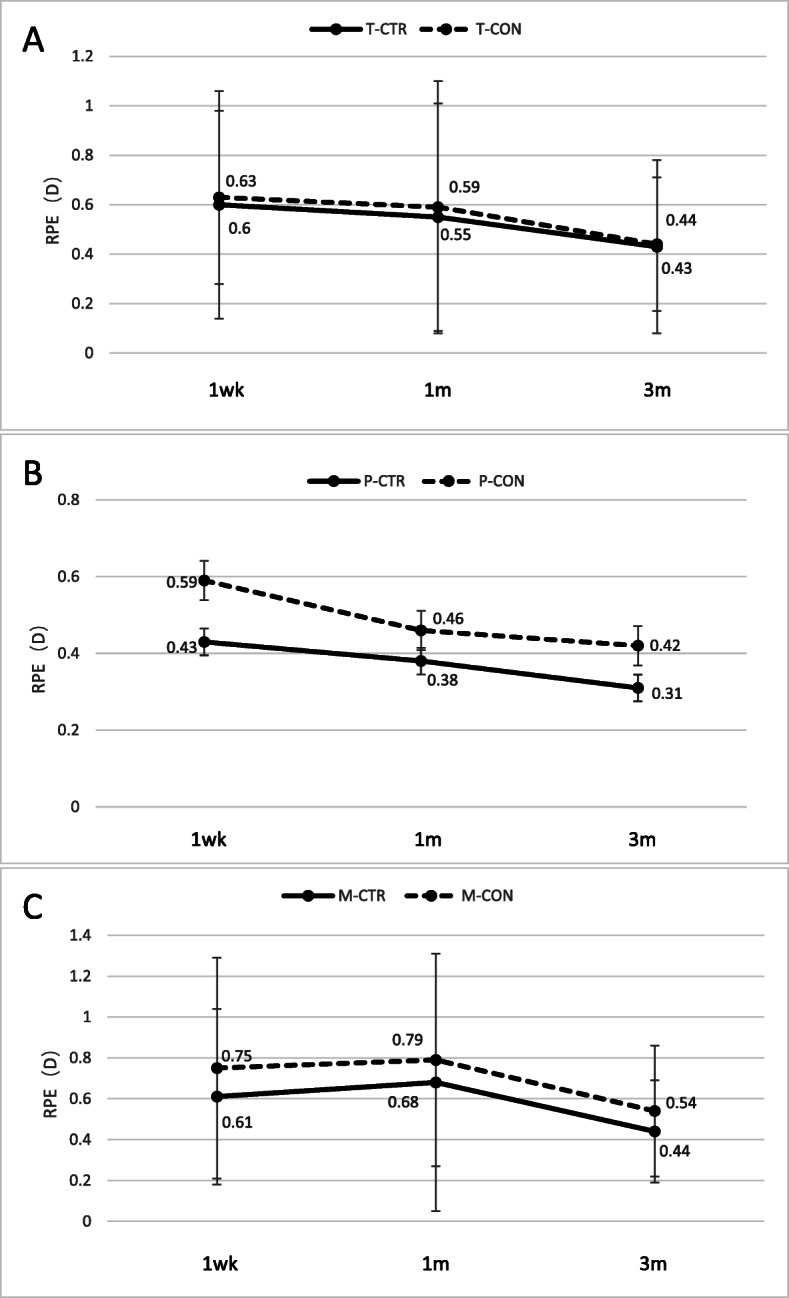
Comparison of refractive prediction error (RPE) at 1 week,1 month, and 3 months after surgery between (**a**) all CTR and control patients, (**b**) the subgroups of CTR and control patients with PPV, and (**c**) the subgroups of CTR and control patients with myopia. T, Total group; P, post-PPV; M, myopia; CTR, capsular tension ring group; CON, control group

Refractive prediction error was not significantly different between CTR and control patients overall (Fig. 3a), or in the subgroup with PPV (Fig. 3b) or strong myopia (Fig. 3c) at any of the time points examined. Data from two patients were discarded because silicone oil was present in the vitreous body.

### Area of continuous curvilinear capsulorhexis (A_CCC_)

A_CCC_ was significantly larger in total CTR patients than in control patients at 3 months after surgery (*p* = 0.0199), but not at 1 week or 1 month (Table 2). Among CTR patients, A_CCC_ was lower at 3 months than at 1 week but not 1 month after surgery. Among control patients, A_CCC_ was lower at 3 months than at 1 week or 1 month after surgery. These findings suggest that CTR implantation can help stabilize A_CCC_ values earlier.

**Table 2:** Area of continuous curvilinear capsulorhexis (A_CCC,_ mm^2^)

Group	n	1 d	1 wk	1 m	3 m
T-CTR	22		22.56±3.61	21.96±3.38	21.53±3.47^b^
T-CON	20		20.70±2.45	20.04 ±2.83	18.76±3.93^bc^
* t*			1.94	1.99	***2.43***
* p*			0.0599	0.0532	***0.0199***
Groups			F = 4.97, *p* = 0.0315		
Time			F = 16.05, *p* < 0.0001		
Group *x* Time			F = 1.85, *p* = 0.1642		
P-CTR	11		21.81±3.58	21.43±3.27	20.58 ±3.09^bc^
P-CON	11		20.82 ±2.49	20.64±2.42	20.22±1.98^b^
* t*			0.75	0.65	0.32
* p*			0.4612	0.5241	0.7494
Groups			F = 0.36, *p* = 0.5557		
Time			F = 8.78, *p* = 0.0007		
Group x Time			F = 1.03, *p* = 0.3665		
M-CTR	11	23.55±0.22*	23.31±3.64	22.49±3.57^b^	22.48±3.71^b^
M-CON	9	23.05±0.43*	20.55±2.56	19.30±3.25^b^	16.97±5.03^bc^
* t*		1.325	1.92	2.07	***2.82***
* p*		0.316	0.0709	0.0529	***0.0113***
Groups			F = 5.86, *p* = 0.0263		
Time			F = 12.44, *p* < 0.0001		
Group *x* Time			F = 5.56, *p* = 0.0079		

*-*n* = 3/group. ^b-^*p <* 0.05 vs. 1 week after surgery within the same group; ^c-^*p* < 0.05 vs. 1 month after surgery within the same group; *T*, Total group, *P* post-PPV, *M* myopia, *CTR* capsular tension ring group, *CON* control group

Further detailed analysis was conducted in subgroups with PPV or myopia. Among CTR and control patients with PPV, A_CCC_ was similar between the two subgroups at 1 week, 1 month, and 3 months after surgery. Among CTR patients with PPV, A_CCC_ was lower at 3 months than at 1 week and 1 month after surgery, while control patients had lower A_CCC_ at 3 months than at 1 week after surgery.

Among CTR and control patients with myopia, while A_CCC_ showed a trend of greater decrease in the control subgroup than in the CTR subgroup from 1 week (*p* = 0.0709) to 1 month (*p* = 0.0529) postoperatively, it was significantly smaller in the control subgroup than in the CTR subgroup at 3 months (*p* = 0.0113) after surgery. Within the CTR subgroup with myopia, A_CCC_ was smaller at 1 and 3 months than at 1 week after surgery, but from 1 month on, A_CCC_ was stable. However, within the control subgroup with myopia, A_CCC_ became smaller with time and was not stable until 3 months after surgery. Moreover, it is noteworthy that when comparing mean A_CCC_ at 1 day to 1 week after surgery in 3 random cases from the CTR subgroup with myopia (23.55 mm^2^) and 3 random cases from the control subgroup with myopia (23.05 mm^2^), A_CCC_ did not seem to start to shrink in the CTR subgroup (23.31 mm^2^) until 1 week after surgery, whereas it showed contraction in the control subgroup (20.55 mm^2^). This suggests an early capsular contraction within 1 week after surgery in patients with severe myopia.

### Ratio of A_CCC_ to area of IOL optics (A_IOL_)

Since we implanted IOLs of two diameters (6.0 or 6.2 mm), the ratio of A_CCC_ to A_IOL_ was also compared between CTR and control groups. When IOL diopter was ≤ + 15.0 D, A_CCC_ was relatively large (5.5–6.0 mm). Similar to the trends in A_CCC_ values, the A_CCC_/A_IOL_ ratio at 3 months after surgery was significantly higher in the CTR group than in the control group (*p* = 0.0172). This trend was also observed in the myopia subgroup (*p* = 0.0124; Table 3).

**Table 3:** Ratio of A_CCC_ / A_IOL_

Group	n	1 wk	1 m	3 m
T-CTR	22	0.77 ±0.13	0.75 ±0.12	0.74±0.12^b^
T-CON	20	0.70±0.09	0.68 ±0.10	0.64 ±0.14^bc^
* t*		1.99	2.01	***2.49***
* p*		0.0529	0.0524	***0.0172***
Groups			F = 5.22, *p* = 0.0277	
Time			F = 16.32, *p* < 0.0001	
Group *x* Time			F = 1.78, *p* = 0.1761	
P-CTR	11	0.77 ±0.13	0.75±0.12	0.72 ±0.11^bc^
P-CON	11	0.72 ±0.10	0.71 ±0.10	0.70 ±0.08^b^
* t*		1.03	0.94	0.68
* P*		0.3144	0.3582	0.5066
Groups			F = 0.83, *p* = 0.3743	
Time			F = 8.6, *p* = 0.0008	
Group x Time			F = 1.06, *p* = 0.3557	
M-CTR	11	0.78±0.13	0.75±0.12	0.75±0.13
M-CON	9	0.69±0.08	0.65±0.10	0.57±0.16^bc^
* t*		1.79	2.03	***2.78***
* P*		0.0908	0.0577	***0.0124***
Groups			F = 5.51, *p* = 0.0306	
Time			F = 12.69, *p* < 0.0001	
Group *x* Time			F = 5.68, *p* = 0.0072	

^b-^*p <* 0.05 vs. 1 week after surgery within the same group; ^c-^*p <* 0.05 vs. 1 month after surgery within the same group; *T* Total group, *P* post-PPV, *M* myopia, *CTR* capsular tension ring group, *CON* control group

### Attachment ratio between posterior lens capsule and IOL surface

Among patients with history of PPV, the attachment ratio between posterior lens capsule and IOL surface was higher in the P-CTR group than in the P-CON group at 1 week and 1 month after surgery (Table 4). The ratio was not significantly different between the M-CTR and M-CON groups at 1 week, 1 month or 3 months after surgery.

**Table 4:** Attachment ratio between posterior lens capsule and IOL surface

Group	n	1 wk	1 m	3 m
T-CTR	22	54.5% (12)	86.4% (19)	90.9% (20)
T-CON	20	20.0% (4)	60.0% (12)	75.0% (15)
* χ*^*2*^		***5.301***	3.767	1.909
* p*		***0.023***	0.055	0.167
P-CTR	11	72.7% (8)	100.0% (11)	100.0% (11)
P-CON	11	27.3% (3)	63.6% (7)	81.8% (9)
* χ*^*2*^		***4.545***	***4.889***	2.200
* p*		***0.043***	***0.045***	0.238
M-CTR	11	36.4% (4)	72.7% (8)	81.8% (9)
M-CON	9	11.1% (1)	55.6% (5)	66.7% (6)
* χ*^*2*^		1.684	0.642	0.606
* p*		0.221	0.370	0.396

*T* Total group, *P* post-PPV, *M* myopia, *CTR* capsular tension ring group, *CON* control group

### IOL inclination angle

**Fig. 4:**
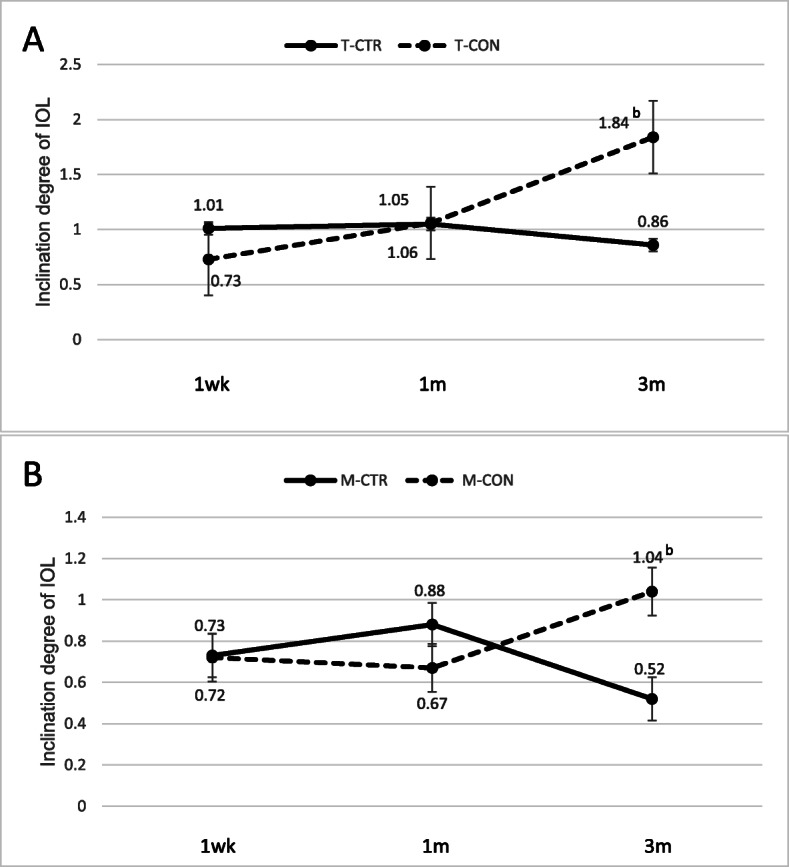
Comparison of intraocular lens vertical inclination angle between (**a**) all CTR and control patients, and (**b**) the subgroups of CTR and control patients with myopia. ^b^- *p* < 0.05 vs. 1 week after surgery within the same group. T, Total group; P, post-PPV; M, myopia; CTR, capsular tension ring group; CON, control group

There were no significant differences in horizontal or vertical IOL inclination angles between CTR and control patients in the total sample or in subgroups with PPV or severe myopia at 1 week, 1 month or 3 months after surgery. Within the total group and within each subgroup, horizontal IOL inclination angle did not vary significantly across the time points from 1 week to 3 months after surgery. Similarly, vertical IOL inclination did not differ significantly across CTR groups (total group and subgroup with PPV or myopia) at any of the postoperative time points. Within the total group of control patients, vertical IOL inclination was significantly higher at 3 months than at 1 week after surgery (0.73 ± 0.46° vs. 1.84 ± 2.15°, *p* = 0.0224; Fig. 4a). Similar results were observed in the subgroup of control patients with myopia (0.74 ± 0.59° vs. 2.82 ± 2.59°, *p* = 0.0286, Fig. 4b).

### Depth of central anterior chamber

Depth of the central anterior chamber did not differ significantly between CTR and control patients in the total sample or in subgroups of those with PPV history or severe myopia at 1 week, 1 month or 3 months after surgery.

### Post-operative complications

All the patients in this study showed well-controlled postoperative inflammation. One CTR patient experienced capsular block syndrome 1 day after surgery. Two CTR patients and one control patient showed transient elevated intraocular pressure (> 21 mmHg). At 3 months after surgery, one CTR patient (4.55%, in the PPV subgroup) and three control patients (15.00%, in the myopia subgroup) showed significant capsular contraction syndrome. No other severe complications were observed.

## Discussion

CTR is widely used in the clinical treatment of cataracts complicated with lens dislocation. CTR can effectively balance the tension of zonular fibers, uniformly distribute the tension of the capsular bag, maintain the shape of the capsular bag, reduce loss of the vitreous body, and increase the attachment between posterior capsule and IOL surface. Therefore, CTR implantation can increase IOL stability after cataract extraction involving IOL implantation and reduce the occurrence of posterior capsule opacification and IOL dislocation [7, 23–35]. However, the benefits of CTR implantation in patients with weakened zonular fibers but no lens dislocation is unclear. In this study, we used a randomized controlled approach to examine the effects of CTR implantation along with phacoemulsification in cataract patients with zonular weakness, in particular because of PPV or severe myopia. Our results suggest that CTR in such patients can reduce the incidence of capsular shrinkage, maintain capsular bag stability, make IOL inclination more manageable and stable, as well as increase the rate of complete attachment between the posterior capsule of the lens and the posterior surface of the IOL.

In cataract patients with lens dislocation, CTR implantation during surgery can lead to better capsular shape and IOL positioning, as well as reduce risk of capsular shrinkage, starting at 1 day up to 6 months after surgery [28]. In that previous study, the capsulorhexis demonstrated a steady trend of contraction from 1 week to 3 months after surgery. The capsulorhexis stabilized after 3 months and showed no significant differences between 3 and 6 months after surgery [28]. Similar results have been observed in humans [37, 38] and animals [39], even in cases without weak zonules. However, other studies found that CTR implantation did not reduce incidence of IOL dislocation at 3 months after cataract surgery [30, 31]. Our results indicated that CTR implantation can prevent capsular shrinkage in patients with severe myopia starting from 1 week after surgery. This effect was even greater at 3 months after surgery, indicating that CTR can effectively delay shrinkage and maintain capsular bag stability during the 3 months after cataract surgery, especially in patients with severe myopia, and this may suggest a possible beneficial effect in the longer term.

In contrast to our findings in patients with myopia, our analysis of patients who had undergone PPV showed no clear beneficial effects of CTR, especially on the anterior capsulorhexis shrinkage. This could be due to the adverse effects of silicone oil or gas bubbles injected into the eye after vitrectomy. Among the 22 PPV patients included in our study, 9 had silicon oil tamponades and 13 had gas tamponades after PPV. Vitreous loss or vitreous filling with gas or silicone oil can change the metabolism of the lens and cause cataracts with capsular plaque and opacification [39]. Thus, the benefits of CTR implantation may be masked by the adverse effects of PPV, silicone oil, or gas bubbles, resulting in reduced capsular elasticity, altered capsule structure, and weakened response to capsulorhexis [40]. Future studies should clarify how silicone oil and gas induce capsular changes.

Capsular contraction syndrome is a serious complication following capsular shrinkage [28, 29], and is usually accompanied by posterior capsule opacification and even causes IOL dislocation. Timely intervention is necessary in order to prevent further visual impairment. In this study, we found that the incidence of significant capsular contraction syndrome was lower in CTR patients (4.55%, 1 case in PPV subgroup, 0 in myopia subgroup) than in control patients (15.00%, 0 in PPV subgroup, 3 cases in myopia subgroup) at 3 months after surgery, which made it mandatory for these patients to have Nd:YAG treatment. This suggests that CTR implantation can reduce, but cannot eliminate, the incidence of capsular shrinkage and contraction, especially in patients with severe myopia [39].

We also found that the rate of complete attachment between the posterior capsule of the lens and the posterior surface of the IOL was higher in the CTR group than the control group at 1 week and 3 months after surgery. This can occur because the CTR mechanically compresses the capsular bag closer to the IOL surface [27, 35, 41, 42]. This effect of CTR was not obvious in patients with severe myopia, perhaps because of their longer axial dimension and larger capsular bags. We speculate that the CTR used in this study, with a diameter of 11 mm, may not be large enough to completely open the capsular bag in patients with severe myopia.

Previous animal and human studies have demonstrated minor IOL eccentricity and inclination that remained stable within 2 years after CTR implantation during cataract surgery [37, 39]. Our study showed that the vertical inclination angle gradually decreased in CTR patients but increased in control patients. In contrast, the horizontal IOL inclination angle remained stable within 3 months after surgery in both CTR and control patients. These results support the theory that IOL inclination after CTR implantation is manageable and stable [23, 43].

Whether CTR implantation during cataract surgery affects postoperative refractive prediction error is unclear. A retrospective study on CTR implantation in 25 patients with abnormal zonules found that the position of the posterior chamber IOL exceeded the predicted value by + 0.5 to + 2.0 D [32]. A randomized controlled trial of 52 cataract patients without other complications showed hyperopia drift after CTR implantation [30], leading those authors to recommend reducing preoperative refractive predictions by 0.5 D. Our results, in contrast, argue against adjusting preoperative refractive predictions for patients undergoing CTR implantation: we did not observe substantial differences in refractive prediction error values between CTR and control patients, consistent with other studies [34, 44–46].

CTR implantation increases the difficulty and risk of cataract surgery, especially in patients who may have hidden capsular rupture. Implanting a CTR may aggravate the rupture of the capsular bag and cause the lens and CTR to fall off. Therefore, surgeons will have a longer learning curve before they achieve proficiency in cataract surgery skills. In addition, the limited adaptations for CTR implantation require special attention in order to prevent severe complications, such as lens and CTR dislocation.

Our results must be interpreted with caution in the light of certain limitations. First, CTR with a diameter of 11 mm was used in all patients, although individualized optimization of CTR diameter is more desirable, especially for cataract patients with severe myopia. Second, due to limited pupil dilation, the IOL profile of certain patients was not fully visible during anterior segment optical coherence tomography. Therefore, only IOL tilt was measured in our study, resulting in inconclusive IOL inclination angle measurements. Third, based on previous studies that have reported the beneficial and adverse effects of CTR implantation, we followed up patients only up to 3 months after surgery. Although we were able to provide some insights into the early beneficial effects of CTR on capsular stability in patients with weak zonules after uneventful cataract surgery, future studies must investigate the long-term stability of the capsule and the effects of CTR on IOL positioning over a long period of time. Further, we could not study the early changes in the anterior continuous curvilinear capsulorhexis (e.g., within 1 week after surgery), since we did not measure A_CCC_ values at 1 day after surgery.

Despite these limitations, our study provides evidence showing that the benefits of CTR implantation can outweigh its disadvantages in cataract patients with slightly abnormal zonules, especially those with severe myopia or those who have undergone PPV.

## Conclusion

CTR seems to be effective in delaying shrinkage and maintaining capsular bag stability in patients with severe myopia, especially in the early stages (3 months) after cataract surgery, indicating possible long-term beneficial effects. However, this early benefit was not observed in patients who had undergone PPV. Our results suggested that CTR implantation may promote adhesion between the posterior capsule and the posterior surface of IOL in patients with weak zonules, especially those after PPV. In a word, CTR implantation can benefit cataract patients with weak zonules by maintaining the shape of the capsular bag, reducing capsule shrinkage and stabilizing IOL inclination, which may reduce risk of IOL dislocation in the longer term. Given the limitations of our study, our findings should be verified and extended in larger randomized studies.

## Data Availability

The datasets used and/or analyzed during the current study available from the corresponding author on reasonable request.

## Abbreviations

CTR: Capsular tension ring
IOL: Intraocular lens
PPV: Pars plana vitrectomy.
UDVA: Uncorrected distant visual acuity
BCDVA: Best corrected distant visual acuity
logMAR: Logarithm of the minimum angle of resolution
RPE: Refractive prediction error
A_CCC_: Area of continuous curvilinear capsulorhexis
A_IOL_: Area of IOL optics
PCO: Posterior capsular opacification
